# Contaminants and Where to Find Them: Microbiological Quality Control in Axenic Animal Facilities

**DOI:** 10.3389/fmicb.2021.709399

**Published:** 2021-08-16

**Authors:** Maria Lebeuf, Nathalie Turgeon, Cynthia Faubert, Alexandre Pleau, Justin Robillard, Éric Paradis, André Marette, Caroline Duchaine

**Affiliations:** ^1^Département de Biochimie, Microbiologie et Bio-Informatique, Université Laval, Quebec City, QC, Canada; ^2^Institut Universitaire de Cardiologie et de Pneumologie de Québec, Quebec City, QC, Canada; ^3^Département de Médecine, Université Laval, Quebec City, QC, Canada; ^4^Pfizer Canada – CIHR Chair in the Pathogenesis of Insulin Resistance and Cardiovascular Diseases, Institut Universitaire de Cardiologie et de Pneumologie de Québec, Quebec City, QC, Canada; ^5^Tier-1 Canada Research Chair in Bioaerosols, Institut Universitaire de Cardiologie et de Pneumologie de Québec, Quebec City, QC, Canada

**Keywords:** germ-free, contaminants, culture, microscopy, quantitative PCR

## Abstract

The use of axenic animal models in experimental research has exponentially grown in the past few years and the most reliable way for confirming their axenic status remains unclear. It is especially the case when using individual ventilated positive-pressure cages such as the Isocage. This type of cage are at a greater risk of contamination and expose animals to a longer handling process leading to more potential stress when opened compared to isolators. The aim of this study was to propose simple ways to detect microbial contaminants with Isocages type isolator resulting by developing, validating and optimizing three different methods (culture, microscopy, and molecular). These three approaches were also tested *in situ* by spiking 21 axenic mice with different microorganisms. Our results suggest that the culture method can be used for feces and surface station (IBS) swabs exclusively (in Brain Heart Infusion for 7 days at 25°C and 37°C in aerobic conditions, and at 30°C in anaerobic conditions), while microscopy (wet mounts) and molecular method (quantitative PCR) were only suitable for fecal matter analyses. *In situ* results suggests that the culture and molecular methods can detect up to 100% of bacterial contamination events while the microscopy approach generates many erroneous results when not performed by a skilled microscopist. *In situ* results also suggest that when an axenic mouse is contaminated by a microbial agent, the microorganism will colonize the mouse to such an extent that detection is obvious in 4 days, in average. This report validates simple but complimentary tests that can be used for optimal detection of contaminants in axenic animal facilities using Isocage type isolators.

## Introduction

Axenic animals are being used more than ever before to study a large variety of subjects since the discovery of the microbiota role on its host. Consequently, one of the most common application for these animals is the use of axenic mice to colonize mice with a known microbiota to study the relationships between the host microbiota and disease. For example, colonization of axenic mice with specific gut microbiota has proved the impact of microbiota on hepatic health (Hartmann et al., 2019); the influence of systemic immune response in some neurological disorders and the impact on pain and cognition in multiple sclerosis, Guillain-Barré Syndrome and Alzheimer’s disease (Catanzaro et al., 2015); and the microbiota implication in chronic intestinal inflammation and in the development of colon neoplasia (Tlaskalova-Hogenova et al., 2011). Axenic mice are also used to study the effect of microbiota on the host physiology which include, for example, regulation of the skeletal muscle mass and function in mice (Lahiri et al., 2019), or induction of obesity (Ridaura et al., 2013).

To maintain the axenic status, animals must be confined to an isolator in order to control their environment and to avoid contamination. Traditional isolators and individually ventilated cages (ICV) are most commonly used. This last type includes the recent positive pressure sealing system, as used by the Tecniplast cage level isolator ISOcage^®^ (Tecniplast^®^, Montreal, Canada, herein referred to as Isocage). Therefore, isolators have considerable disadvantages. For example, all the animals contained in the isolators share the same air, meaning all axenic animals in a given isolator are at risk of losing their axenic status in the event of a contamination (Hecht et al., 2014; Paik et al., 2015; Basic and Bleich, 2019; Lange et al., 2019; Niimi and Takahashi, 2019; Niimi et al., 2019). At the opposite, all Isocages are their own isolator at cage level and can allow multiple simultaneous protocols at a time in the same rack. However, despite these cages being their own individual hygienic units (Brielmeier et al., 2006), each cage is still at risk of being contaminated by the outside environment every time it is opened.

Microbiological quality controls for axenic animals housed in traditional isolator or cage level isolator almost use the same methodology. Exception is that in cage level isolator, each of them must have its own microbiological status confirmed. In general, a mix between culture, molecular techniques and microscopy approaches is applied to define the axenic status of animals (Schoeb et al., 2017). For culture methods, feces, bedding, and food are the most frequently used samples. Samples are incubated in two or more culture media, in aerobic and anaerobic conditions, at a single or multiples temperatures (Arvidsson et al., 2012; Hecht et al., 2014; Fontaine et al., 2015; Karaman, 2015; Nicklas et al., 2015; Paik et al., 2015; Basic and Bleich, 2019; Lange et al., 2019; Niimi et al., 2019). The liquid and solid culture media used vary a lot. Although, the two more often chosen are the brain heart infusion medium for the isolation of bacteria (Arvidsson et al., 2012; Nicklas et al., 2015; Niimi et al., 2019) and the thioglycolate medium for the isolation of fungi. Incubation time varies between 24 h and 14 days, but 7 days is the most frequently chosen delay (Arvidsson et al., 2012; Hecht et al., 2014; Fontaine et al., 2015; Nicklas et al., 2015; Paik et al., 2015; Lange et al., 2019; Niimi et al., 2019). However, although culture approach is simple and inexpensive, it has limitations in terms of nutrient requirements or in the growth rate of certain microorganisms, which can lead to erroneous results (Fontaine et al., 2015). Culture media are easy to contaminate and false positives can generate significant financial and animal losses (Fontaine et al., 2015). Furthermore, the chances of contamination increase if the sample has to undergo a lot of processing before incubation. According to the literature concerning Isocage quality control, samples are collected and tested either for each cage individually (Hecht et al., 2014) or are pooled for group analysis (Niimi et al., 2019). However, this latter way of proceeding, although it requires less investment than individual sampling, does not make it possible to target the faulty cage in the event of positive results. In addition, the longer the cages are open to collect samples, the longer the animals are exposed to outer cage environment. Moreover, and the longer their circadian cycle is compromised, which can cause stress and affect protocols results.

For microscopy in axenic context, wet mounts are sometime selected, but Gram strains remain the most used method for the determination of axenic status (Schoeb et al., 2017). Observations of feces and/or cecal contents of animals are performed at 1000× magnification (Fontaine et al., 2015; Schoeb et al., 2017). Readings are gathered with immersion oil by diluting the sample in sterile water or saline (Schoeb et al., 2017) or by direct observation of a swabbed feces (Fontaine et al., 2015). However, it has been demonstrated in the literature that bacteria present in food before autoclaving or after irradiation can be detected by microscopy (Taylor et al., 1986; Midtvedt and Gustafsson, 2005; Fontaine et al., 2015; Nicklas et al., 2015; Niimi et al., 2019). Nevertheless, bacteria retain their morphology even after passing through the digestive tract and keep the possibility of absorbing dye (Taylor et al., 1986). Although they are not detectable by molecular methods due to the degradation of their DNA (Midtvedt and Gustafsson, 2005; Fontaine et al., 2015; Nicklas et al., 2015; Niimi et al., 2019). Consequently, this complicates the analysis when the objective is to demonstrate that an animal is free from microorganisms. It is often advisable to interpret microscopy results in parallel with culture and molecular method results (Schoeb et al., 2017). It is also recommended to use the expertise of an experienced microscopist since mastering microscopy techniques requires some practice (Packey et al., 2013; Schoeb et al., 2017). It has been documented that some of the authors also proposed the detection of parasites using microscopy approaches (Karaman, 2015; Nicklas et al., 2015).

Finally, the use of molecular methods has the advantage of allowing the detection of microorganisms that are not culturable (Schoeb et al., 2017). Furthermore, it also permits detection of fastidiously culturable bacteria that could colonize axenic animals (Schoeb et al., 2017). Even though good methods for characterization of microbial populations are used in several fields in microbiology, such as polymerase chain reaction denaturing gel electrophoresis (PCR-DGGE) (Blais Lecours et al., 2012; Just et al., 2013; El Sheikha, 2019), non-quantitative, and quantitative polymerase chain reactions (PCR and qPCR) are most used for detecting bacterial contaminants in axenic animals (Arvidsson et al., 2012; Packey et al., 2013; Fontaine et al., 2015; Nicklas et al., 2015; Paik et al., 2015; Basic and Bleich, 2019; Lange et al., 2019). These reactions are performed by using primers targeting the conserved region of the 16s rRNA gene (Arvidsson et al., 2012; Packey et al., 2013; Fontaine et al., 2015; Nicklas et al., 2015; Paik et al., 2015; Basic and Bleich, 2019; Lange et al., 2019). The use of this type of universal primer is optimal for the detection of bacteria since the identity of the contaminants is unknown. Mice feces are the most often used sample, treated with extraction kits specially designed for feces (Fontaine et al., 2015; Nicklas et al., 2015; Paik et al., 2015; Lange et al., 2019). In addition, extreme care must be taken when processing axenic samples used for molecular method. Indeed, all microorganisms entering in the samples can potentially cause amplification, and therefore false positive. Consequently, molecular method carry a significant risk of contamination (Nicklas et al., 2015). Next generation sequencing allows the detection of the complete microbiome in a sample and does not rely on specific primers. However, samples with low DNA concentration can also generate false results and confound analysis (Robin et al., 2016). Once again, since axenic mice samples are supposed to contain no or very few microorganisms, they are considered low DNA concentrated. These drawbacks are therefore not optimal for a routine quality control program, which is wanted to be simple, to give accurate results and avoid undesirable animal and monetary losses. It should be kept in mind that molecular methods, unlike culture and microscopy, do not allow the detection of several types of microorganisms and are rather specific. This means that several reactions with different primers targeting variety of microorganisms must be performed in order to obtain information on the likely contaminants of an animal. It has thus been documented that the presence of other microorganisms than bacteria is sometimes sought by molecular method i.e., for *Archaea* and molds (Packey et al., 2013) and certain specific viruses (Nicklas et al., 2015). However, a recent publication has demonstrated that bacteria from mammal skin and gut, as well as environmental microorganisms can be found on axenic room cages (Lebeuf et al., 2021).

There is a wide range of methods that are currently used to detect a microbial contamination in axenic conditions. These go from those that target only a single microbe to those that target multiple microbial types. Methods used for the sampling and the sample choice of cage level isolators such as Isocage also tend to be extremely varied. This is due the fact that there is no consensus about the best way to detect a contamination. The majority of the detection methods are vulnerable to post-sampling contaminations since they require samples to be handled after they leave the biosafety station. Furthermore, the large number of cage samples that are collected for the cultures increase the amount of handling required, in addition to being time-consuming. On the other hand, microscopy methods require a minimum of microscopy skills. Otherwise, a longer handling time of Isocages in IBS when performing cage changing can be stressful for animals and could have an impact on results of protocols using axenic mice.

The aim of this study was to determine the more effective methods for detecting a bacterial contamination using the Isocages. This was achieved by validating and optimizing three different methods (culture, microscopy, and molecular) and by challenging the final optimized methods *in situ* by spiking axenic animals with different microorganisms.

## Materials and Methods

The protocols presented below were design and prepared before the beginning of the study. This includes elaboration and optimization of three different methods and the use of their optimized form for *in situ* tests with mice. The protocol was approved by ethic committee before beginning of experimentation with animals.

### Axenic Animal Facility

The axenic animal facility used in this report consisted of two rooms that are exclusively used for axenic or gnotobiotic purposes, as previously described by Lebeuf et al. (2021). Each room contained a full rack of 30 Isocages and an Isocage Biosafety Station (IBS, Tecniplast^®^). The IBS was equipped with a disinfectant dunk tank in which all incoming and outgoing material transited through to avoid laminar airflow perturbations. Only the incoming material was disinfected using a 200 ppm MB-10 solution (Quip Laboratories, Wilmington, NC, United States), while a spray of the same solution and concentration was used between cage handling on the work surface and gloves inside de IBS. Between each utilization, the inside of the IBS was fumigated for 3 h with 30% oxygen peroxide (J. T. Baker^®^, Avantor^®^, Radnor Township, PA, United States). The entire room was also fumigated with the same gas every 3 months. There was an antechamber attached to each of the two rooms, allowing for material and staff preparation. Personnel that handled the animals were required to follow a personal protection protocol. Before entering the room: wash hands and put on medical gloves, a hair cover, a coverall suit, a full-face chemical cartridge mask (Powered air-purifying respirator, 3M Health Care, Saint Paul, MN, United States), clean slippers and a then second pair of gloves. For daily animal observations which did not need cage opening, a single-use medical gown and N95 mask (3M Heath Care) were used instead of the coverall suit and full-face chemical cartridge mask.

When performing cage changes, Isocages containing the animals and clean cages passed through the dunk tank for a 5-min disinfection process, then introduced in the IBS. To minimize animal stress and risks of contamination, mice where immediately transferred to clean cages using sterile forceps (less than 10 s) and the cages were closed. The clean cages containing animals were taken out of the IBS through the dunk tank and placed back in the racks while the technician proceeded to sample collection for microbiological quality control.

### Cultures

The samples tested for the culture methods were collected from the direct environment of the mice (all the elements present in the cage, feces, bedding, water, food, and cotton wool called Nestlet). Swabs from the Isocage Biosafety Station (IBS) were also collected in order to ensure a sterile work surface. The culture medium used was Bacto^TM^ Brain Heart Infusion (BHI, BD, Sparks, MD, United States). The samples were introduced directly from the cage to the BHI tubes inside the IBS. Three different incubation conditions were applied to screen for as many types of microorganisms as possible (25°C aerobic for environmental microorganism, 37°C aerobic for human and mice colonizers, and 30°C for anaerobic microorganisms, 30°C allowing environmental and mice colonizers bacteria, 25°C was not needed since no fungi can grow in anaerobic condition). Samples were incubated for up to 7 days to allow for the detection of slow growing microorganisms. At the end of the incubation period, medium turbidity was measured using an optical density (OD) reader (Cell Density Meter Model 40, Thermo Fisher Scientific, Waltham, MA, United States). Samples with an OD greater than zero were considered to be potentially positive for contaminants. In these cases, isolation measures were put in place, in which samples were directly examined using phase-contrast microscopy and subcultured on solid Bacto^TM^ Brain Heart Infusion Agar (BHIA, BD) medium. The samples were considered positive if bacteria were visible under a microscope or in the subcultures.

#### Validity, Confirmation, and Optimization of the Culture Method

In order to determine the most sensitive sample types for contaminant detection in Isocages, non-axenic mice were introduced individually into sterile cages. This was done to assess the likelihood of the mice to contaminate elements of their environment during the time between two cage changes. Samples of the soiled cages were collected at every cage change for 6 weeks for a total of 108 Isocages changed in March and April 2017. The contamination profile of the non-axenic mice was then validated by comparing with the positive samples. These positive samples resulted from four occasional and accidental contaminations of seven Isocages hosting axenic animals that occurred during the first year of the axenic platform (June 2017 to November 2018). Finally, optimization of the culture method was obtained by targeting the highest percentage of positive samples. This percentage was obtained with either method from both axenic and non-axenic mice using a Tukey’s multiple comparison test. The results were considered significant when *p-values* were less than 0.05.

### Microscopy

The microscopy method used in this research involved direct microscopic observations of samples. Cage samples (feces, bedding, water, food, and cotton wool called Nestlet) from three types of mice were selected. Mice types were: non-axenic mice (NA, 15 cages); accidentally contaminated axenic mice with negative culture (AC, 4 cages); and axenic mice (A, 7 cages). All samples were collected in triplicate and diluted in 1 mL of sterile water. Observations were conducted by examining 20 near-fields of a sample for a maximum of 10 s each using Laborlux S Microscope (Leitz, Stuttgart, Germany). Blind readings using wet mounts and Gram stains were performed for all samples.

### Molecular Method

Molecular methods were tested on fecal samples from axenic mice (15), non-axenic mice (4) and accidentally contaminated mice (7). DNA extraction was performed on five mice feces using the QIAamp^®^ PowerFecal^®^ DNA kit (Qiagen, Hilden, Germany) in sterile conditions. A real-time polymerase chain reaction (qPCR) was performed using a CFX96 Touch Real-Time PCR Detection System (Bio-Rad, Hercules, CA, United States) on the samples according to the amplification protocol described by Bach et al. (2002), but with a Mastermix total reaction of 20μL instead of 50μL. The universal total bacteria primers EUB R (5′-GACARCCATGCASCACCTG-3′) and EUB F (5′-GGTAGTCYAYGCMSTAAACG-3′) were used to determine the total quantity of bacterial DNA in each sample (Bach et al., 2002). A standard curve was used to determine bacterial concentration in feces by using 1E+01 to 10E+06 copy of *Escherichia coli* genomic DNA. Consequently, the total bacteria concentration in samples was expressed in *E. coli* equivalent genomes per gram of feces. Negative controls containing no template were run for the extraction and qPCR.

#### Data Analysis

The Bio-Rad CFX Manager software (Version 3.1) was used to analyze the melting curve. The Protocols for determination of limits of detection and limits of quantitation; approved guideline» by the Clinical and Laboratory Standards Institute (Armbruster and Pry, 2008) were used to determine the limit of blank (LOB, Equation 1) and the limit of detection (LOD, Equation 2) for the molecular methods used in this study.

(1) $$\left(\text{LOB}=\mu_{B}+1.645\sigma_{B}\right)$$

(2) $$\left(\text{LOD}=\text{LOB}+1.645\left({\text{SD}}_{Lowconcentrationsample}\right)\right)$$

The LOB was used to assess the background noise generated by the presence of contaminant DNA introduced during sample handling. The LOB was based on the mean of negative controls (μ_*B*_) (extraction control and qPCR no template control) and the standard deviation of the negative controls (σ_*B*_). By using the LOD, it is possible to determine the threshold at which a sample can be considered positive. The LOD is based on the LOB and the standard deviation between samples of known low concentrations (SD_*Low concentration sample*_). In this study, the standard deviation of samples containing 10 copies of genomes was used. The LOB and the LOD were determined for every extraction and qPCR analysis.

### *In situ* Testing of Methods Effectiveness for Detection of Spiked Microbial Contamination Over Time

#### Animals

Twenty-one six-weeks-old male C57BL/6 axenic mice from Charles River Laboratories (Wilmington, MA, United States) were used and housed in as many Isocages for this experiment. All the mice were handled according to the CPAUL and the CCPA guidelines and as described in the ethics authorization request # 2016-109-4A before the protocol started. Sets of three axenic mice were artificially contaminated with only one of the following non-pathogenic microbial strains: *Staphyloccocus epidermidis* (SE, 37°C aerobic, isolated from a previously contaminated axenic Isocage, GenBank sequence ID: MT585538); *Lactobacillus reuteri* (LR, 37°C anaerobic, isolated from a non-axenic mouse feces, GenBank sequence ID: CP054657.1); *Bacillus atrophaeus* (BA, 25°C aerobic, from the author’s bacterial collection and previously used as a model for the management of contaminants in axenic mice facilities (Lebeuf et al., 2021), GenBank sequence ID: MN826517.1); *Clostridium sporogenes* (CS, spore forming anaerobic bacterium, mice gut colonizer, often used in germ-free mice researches (Itoh and Mitsuoka, 1985; Furusawa et al., 2013), from Luc Trudel at Université Laval, GenBank sequence ID: MT356160.1); or *Saccharomyces cerevisiae* (SC, to act as a negative bacterial control, common mice gut colonizer (Clark, 2017), also to provide data about yeast detection in axenic context, isolated from Fleichmann’s instant yeast, GenBank sequence ID: NR_111007.1). Three axenic mice were maintained as negative controls. As presented in Figure 2A, a pellet was contaminated with approximately 100 cells of a single microorganism and put in the cage for 24 h. This approach was chosen to serve as a contamination source mimicking a natural contamination event. After this period, cages were changed and samples were collected. Additional samples were collected after 4, 7, 11, and 14 days to determine the incubation period required to detect the microorganisms that were introduced.

#### Cultures

Even if the optimized version of the culture method involved three tubes incubated (BHI broth at either 25°C and 37°C in aerobic conditions, and at 30°C in anaerobic conditions) a total of 19 fecal samples were collected per cage and were incubated for statistical purpose. If, for example, a bacterium that can only grow in anaerobic condition, there is a theoretical 30% chance that the contamination can be detected through the culture method. However, if a non-contaminated faece is selected, the results could be 0% and the animal might be declared as negative, even if it was contaminated. To have an idea about the chance to pick a faece containing bacteria when performing a routine bacterial screen, more samples were cultured in the optimal growth condition (C_*x*_) of each targeted microorganism amongst the three conditions of the culture method (37°C aerobic, 25°C aerobic, or 30°C anaerobic). For example, L. reuteri (LR) being an anaerobic bacterium, 17 of the 19 fecal samples were incubated under 30°C anaerobic, which is its optimal condition (C_*x*_), while the two remaining were incubated in 37°C and 25°C aerobic (C_*A*_ and C_*B*_). Therefore, the average obtained from Cx would provide an overview of the chances of selecting a positive sample in the cage if LR grow in anaerobic condition exclusively. The likelihood of detecting spiked microbial contamination when testing the culture method effectiveness *in situ*, expressed in percentage of positive samples (%Pos), was calculated using Equation 3.

(3) $$\left(\%\mathrm{Pos}=\frac{\left(C_{A}+C_{B}+C_{x}/17\right)}{3}\right)$$

The protocol for this experiment has been slightly modified and repeated for the microorganisms that did not successfully colonize the mice within 24 h. Instead of conducting a cage change after 24 h post-contamination, mice were kept in the same cage for 2 weeks, with feces collection after 24 h, 4, 7, and 14 days.

#### Microscopy

For microscopy analyses, wet mounts of a fresh feces from each cage, diluted in 1mL of sterile water, were used and examined by four microbiologists: three with basic knowledge in microscopy (basic microscopists), and a skilled microscopist. Microscopists had to indicate the morphology of the seen microbial contaminants as well as their quantity for 20 near-fields at 1000× magnification. All three basic microscopist results were compared to the skilled microscopist to verify the reading accuracy according to the level of knowledge in microscopy. For the data analysis, false positive means that the feces sample was considered positive when the mice were not contaminated or when microscopist noted another type of microorganism morphology (e.g., noted positive with cocci while animals were contaminated with *Bacillus*). False negative was when a mouse was contaminated, but the presence of microorganisms was not noted by a microscopist. Unconclusive results mean that the microscopist was not able to unequivocally categorize the mice status according to the microscopy method only. The animal status was determined according to the other two methods and to the skilled microscopists lectures.

#### Molecular Method

For the detection of the spiked microorganisms in mice feces, the same optimized quantitative PCR as previously described in the section *“*Materials and Methods” and section “Molecular Method” was used, including the data analysis procedure. The identification of each microorganism used for artificial contamination was confirmed using 16S sequencing from samples collected on day 0 (contamination solution) and day 14 post-contamination (isolated from feces) as follow. DNA extraction was performed using a Whatman^TM^FTA^TM^ Classic Card (GE Healthcare Life Science, now called Cytiva, Marlborough, MA, United States), as recommended by the supplier. A PCR was performed for each using 5 μL of *5*× *Green GoTaq Flexi buffer*, 1 μL of *dNTP mix*, 3 μL of *MgCl*_2_, 0.2 μL of *GoTaq Flexi Polymerase* (Promega, Madison, WI, United States). To identity bacteria, 50 mM of universal 16S RNA primers 63F (5′-CGGCCTAACACATGCAAGTC-3′) and 1387R (5′-GGCGGWGTGTACAAGGC-3′) were used (Marchesi et al., 1998). ITS region primers ITS1 (5′-TCCGTAGGTGAACCTGCGG-3′) and ITS4 (5′-TCCTCCGCTTATTGATATGC-3′) were used for yeast identification in the same concentrations as for the bacterial primers. Three punches from FTA^TM^ cards were incorporated into the final 50 μL PCR reaction for all strains. Amplifications were performed using a thermocycler (DYAD Peltier thermal cycler, Bio-Rad), and consisted of a 4 min 30 s of activation at 94°C, followed by total of 40 cycles including at 94°C for 30 s, 58°C for 30 s, 72°C for 90 s. A final extension was performed at 72°C for 5 min for the bacteria, while the protocol described by White et al. (1990) was performed for the yeast.

## Results

### Cultures

**FIGURE 1:**
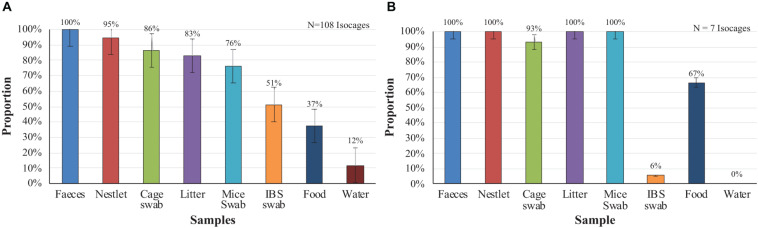
Proportion of positive samples per culture when non-axenic and accidentally contaminated axenic mice are introduced into sterile cages after two weeks. **(A)** Non-axenic mice. **(B)** Accidentally contaminated mice.

The results from the introduction of contaminated mice into sterile cages are presented in Figure 1A. In 100% of the 108 Isocages, the feces showed microbial growth, followed by Nestlets (95%), cage swabs (86%), and bedding (83%). The percentages of positive samples for mouse swabs, IBS swabs, food, and water were 76, 51, 37, and 12%, respectively. For the occasional and accidental contamination of axenic mice (Figure 1B) a similar pattern of contamination was observed. One-hundred percent of the feces, Nestlets, bedding and mouse swabs were positive, while smaller percentage of cage swabs (93%), IBS swabs (6%), food (67%), and water (0%) were positive. However, the samples occasionally generated ODs greater than zero even though there was no deposit or macroscopic sign of growth before agitation of the tubes. There were no colonies detected on solid media and there were no visible microorganisms identified through microscopy. These samples were therefore considered to be negatives. The lowest positive OD that was observed was 0.35. Statistical analyses showed no significant differences between feces and Nestlet, litter and mice swab for the non-axenic mice, while significant differences were found for the other samples. For the accidentally contaminated axenic mice.

### Microscopy

Microscopy results are presented in Table 1. For bedding and water, all the samples were considered negative for the three mouse types and the two microscopic methods. For food and nestlet, unconclusive and negative results were mostly obtained from all mouse types. An unconclusive result means that the microscopist was not able to unequivocally confirm the status of the sample due to the presence of indistinct elements on strains during readings. The water samples contained no detectable bacteria in all of the samples from both microscopic treatments. Feces samples from non-axenic mice (NA) were positive and axenic mice (A) were negative using both treatments. Finally, for the axenic accidentally contaminated mice (AC), feces were considered positive using wet mount while unconclusive with Gram strain.

**TABLE 1:** Blind readings of Isocage samples using wet mounts and Gram stain for different mouse types.

Test	Mouse type	Feces	Bedding	Nestlet	Food	Water
Wet mount	NA	**+**	**−**	+/**−**	+/**−**	**−**
	AC	**+**	**−**	+/**−**	**−**	**−**
	A	**−**	**−**	+/**−**	**−**	**−**
Gram stain	NA	+	**−**	+/**−**	+/**−**	**−**
	AC	+/**−**	**−**	**−**	+/**−**	**−**
	A	**−**	**−**	**−**	+/**−**	**−**

### Molecular Method

No trace of DNA was detected in axenic mouse feces. On the other hand, an average of 8.63E+10 *E. coli*-equivalent genomes per gram of feces (EqGen/g) in non-axenic mice. Finally, 7E+09 EqGen/g was detected in accidentally contaminated axenic mice.

### *In situ* Testing of Methods Effectiveness for Detection of Spiked Microbial Contamination Over Time

#### Cultures

**FIGURE 2:**
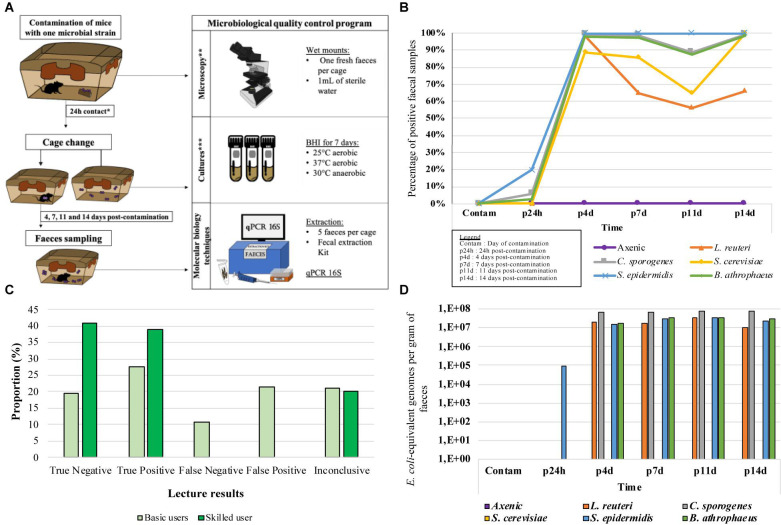
*In situ* protocol and results for the three optimized methods. **(A)** *In situ* protocol. *24h post-contamination cage change was not applicable for the second attempt only, applicable to 2 GF, 3 LR, 3 SC, and 2 BA. **3 basic and 1 skilled microscopists performed the blind wet mounts lectures to determine microscopy method effectiveness *in situ*. ***Exceptionally for the *in situ* protocol, 19 samples were taken for the culture method. 17 tubes were incubated in the microorganism’s optimal condition, and the two other tubes were incubated in the two other conditions. **(B)** Likelihood of detecting spiked microbial contamination when testing the culture method effectiveness *in situ*, expressed in percentage of positive samples. **(C)** Proportions of the 5 types of results obtained from 4 microscopists during the examination of faeces sample from microbially spiked mice in order to test the effectiveness of the microscopic method *in situ.* **(D)** DNA concentrations of each organism found in faces of spiked mouse in order to test the effectiveness of the molecular method *in situ.*

After 14 days in the cages, the negative control axenic (A) mice remained axenic according to culture method. All IBS swabs showed no growth during all the protocol. For culture of feces from mice contaminated with *Clostridium sporogenes* (CS) and *Staphylococcus epidermidis* (SE), bacterial presence was detectable 24 h post-contamination (p24h) in 3/19 feces and 10/19 feces, respectively, on average. After 14 days (p14d), all the feces collected were positive for CS and SE. Only one out of the three cages contaminated with *Bacillus atrophaeus* (BA) showed growth in feces between 24 h (4/19 samples) and 14 days (19/19 samples). For the two other BA-contaminated cages as well as the cages contaminated with *Lactobacillus reuteri* (LR) and *Saccharomyces cerevisiae* (SC)-contaminated cages, none were positive from the day of contamination to 14 days post-contamination. The results demonstrated growth for all of the strains after 4 days (p4d; 18/19 feces for LR and BA, 19/19 feces for SC) and 14 days (p14d; 17/19 for LR and 17/17 for BA and SC). After 14 days, all of the known bacterial strain identities were confirmed in the samples. According to the Equation 3 and as presented in Figure 2B, the likelihood for the detection of a microbial contamination after 14 days varied between 66 and 100%, according to the used microbial models (66% for LR, 100% for SE, BA, CS, and SC). Note that only the cages that showed positive growth have been used to produce the Figure 2B, except for the negative controls.

#### Microscopy

Using microscopy, fresh feces from each cage were observed on wet mounts by three microbiologists with basic knowledge in microscopy (basic microscopists), as well as a skilled microscopist. On many occasions (18/56), basic microscopists falsely identified contaminants in feces samples of the axenic mice. On the other hand, the skilled microscopist generated no false positives (0/28), and 4 unconclusive results (4/28). At p24h, 9/22 axenic feces were falsely considered positive while 4/6 were accurately identified as positive for CA and BA by the basic microscopists. None of the SE cages were noted positive by the basic microscopists. For the skilled microscopist, 2/3 CS and 1/3 SE has been positive while 1/3 CS and 2/3 SE were unconclusive. Microorganisms were detectable by all four microscopists between p4d and p14d. However, LR was detected for 9/24 (pooled results) feces by basic users compared to 11/12 feces for the skilled user. For all 161 microscopic observations made during the experiments (Figure 2C), the basic microscopists correctly identified an average of 38 to 51% of the samples, while 22to 44% of the samples were erroneous results (false positives or false negatives). For the skilled microscopist, 80% of the results were correctly identified, with no erroneous results. However, in 20% of cases, the skilled microscopist was unable to determine the result. All the *Saccharomyces cereviseae* (SC) samples were accurately identified by the skilled microscopist, while only one of the three basic users detected this strain once throughout the entire protocol.

#### Molecular Method

Using molecular method, none of the feces from the axenic (A) mice or the mice contaminated with yeast (SC) contained bacterial DNA after amplification (Figure 2D). After 24 h, *S. epidemidis* (SE) was the only strain of detectable bacteria, with a concentration of 9.1E+04 *E. coli*-equivalent genomes per gram of feces (EqGen/g). For all the other strains, concentrations between 1.06E+07 and 7.83E+07 EqGen/g were detected from 4 days post-contamination (p4d) to 14 days post-contamination (p14d). One-hundred percent of the model bacteria were detectable from p4d, while 0% of the yeasts were detected using this method. However, the yeasts were detected using the ITS primers mentioned previously.

## Discussion

### Cultures

For both the non-axenic mice that were introduced into sterile cages (Figure 1A), and the axenic mice that were accidentally contaminated (Figure 1B), feces tested positive for contamination 100% of the time. In contrast, water samples had the lowest proportion of positive samples. It can be perhaps due to the low levels of nutrients available in the drinking water and the tightness of seal between the straw and the cap, reducing chances for bacterial intrusion. For the IBS swabs, the sampling campaign in which accidentally contaminated axenic mice were involved having took place months after the one using non-axenic mice. The lowest proportion of positive samples could demonstrate an improvement of the staff in axenic mice handling.

It is time consuming to collect samples from all the materials (feces, food, Nestlet, water, bedding, and swab) in all the cages in triplicate and incubate the samples under three different conditions. Furthermore, the longer it takes to collect samples, the longer the mice circadian cycle is compromised by the cage changing process noises. Therefore, in order to reduce the time burden, only the sample type that most frequently tested positive was selected for the regular quality control procedures. Fecal samples were the only sample that had 100% of detection effectiveness for both non-axenic (Figure 1A) and accidentally contaminated axenic (Figure 1B) cages when cultured. The IBS swabs, despite their low percentage of detection (54% for non-axenic, 6% for axenic accidentally contaminated), were conserved as control for technical handling. Since some samples tended to generate ODs that were greater than zero even without contamination and the presence of microbial growth in tubes were visually evident, ODs were not preserve for regular quality control procedures. The three incubation conditions having their proper microorganism targets (i.e., 25°C aerobic for environmental microorganism, 37°C aerobic for human and mice colonizers, and anaerobic at 30°C) none of them was eliminated. The choice of brain heart infusion (BHI) as unique culture media was encouraged for simplicity and efficiency concerns. BHI being a non-selective medium allowing fastidious and non-fastidious bacteria, yeast, and fungi, it represents a good polyvalent compromise between several of the media used by other axenic users. However, knowing that a great part of axenic environment are colonizers of the human, other mice hosted in axenic facility and environmental microorganisms (Basic and Bleich, 2019; Lebeuf et al., 2021), the choice of media and incubation conditions are adapted to most probable contaminant of axenic cages. Consequently, knowing that some microorganisms might not growth in this media, the use of other methods of detection is recommended to detect them.

### Microscopy

The material fibers in the bedding and food samples caused a great deal of refraction on wet mounts and absorbed a large amount of Gram dye. This made it extremely difficult to distinguish microorganisms and occasionally led to inconclusive readings. The same was true for the Nestlet, which also produced the cotton fibers that made detection more challenging and sometimes led to distorted images of the specimens under the microscope. Water samples contained such a diluted number of microorganisms that they sometimes could not be detected in the non-axenic (NA) mouse cages. Discerning the microbiological status from fecal samples was easier when using wet mounts, which allowed for the visualization of microbial motility, as reported elsewhere (Nicklas et al., 2015). Observations using Gram straining was at times arduous to perform these types of observations and is therefore recommended, as previously mentioned (Arvidsson et al., 2012; Packey et al., 2013; Fontaine et al., 2015; Karaman, 2015).

### Molecular Method

No microbial DNA was observed in axenic mouse feces, as previously noted (Packey et al., 2013; Fontaine et al., 2015; Paik et al., 2015). For the accidentally contaminated axenic mice, 7E+09 EqGen/g was detected. This value is higher than what was observed elsewhere with contaminated mice: 10E+05 to 10E+06 copies of the 16S rRNA gene per gram of feces (Packey et al., 2013). There was an average of 8.63E+10 EqGen/g detected in non-axenic mice, suggesting that a high concentration of DNA could be present when a contamination occurs. However, there is no proof of a naturally low concentrations contaminants in axenic contexts. This suggests that either modest contamination concentrations are not possible in axenic animals, or this kind of contamination has never truly been detected due to the limitations in the methods that are currently used. This was supported by Fontaine and collaborators who showed that low spiked concentrations of contaminants (under to 10E+03 to 10E+4 colony forming-units per gram of feces) could not be detected by PCR (Fontaine et al., 2015). However, the literature also suggests that in a niche where there are no biological habitants (here, the mice and the cage), the first compatible colonizer to arrive (here, microorganisms) will often show great dispersal capacity in the unoccupied space, especially if the environment responds to the microorganism’s nutritional needs (Atlas et al., 1998; McArthur, 2006).

### *In situ* Testing of Methods Effectiveness for Detection of Spiked Microbial Contamination Over Time

#### Cultures

Optimized culture method results (Figure 2B) demonstrated that as quickly as 24 h post-contamination (p24h), certain contaminants could be detected in axenic cages (SE, CS, and BA), while others required more time to become detectable (LR, SE, and BA). This result suggests that for some of the microorganism, more than 24 h may be required to colonize an animal. For almost all the strains, there was a high level of positive detection between p4d and p14d. Considering that all fecal samples were retained in the cages from day 0 to day 14, it is possible that the feces from when the animals were axenic (before contamination) or fecal samples containing dead bacteria were used for the culture and resulted in negative reads. The results also show that in 66% to 100% of cases, contamination was detectable using only three fecal samples that were incubated for a maximum of 7 days in BHI (at 25°C and 37°C in aerobic conditions and at 30°C in anaerobic conditions). However, since there remains a possibility of contamination with unculturable microorganisms in the selected incubation conditions, we recommend the use of other methods, such as microscopy and molecular method to complement the culture method and to confirm axenic status.

#### Microscopy

For microscopy using wet mounts and fresh feces, the accuracy of the results was determined by the microbial concentration, the microorganism morphology and the microscopist’s level of experience (22 to 44% error for microscopists with basic knowledge versus 0% for the skilled microscopist). All the axenic samples and those from p24h were the most inaccurately observed by the basic-level microscope users, since they contained none or very few microorganisms.

#### Molecular Method

For the molecular method, only *Staphylococcus epidermidis* (SE) was detected at p24h, contrary to what was observed using cultures and microscopy (CS and BA). However, CS and BA demonstrated low bacterial concentrations, which can affect detection. The high DNA concentrations that were detected between p4d and p14d suggest once again that high concentrations of DNA may be present when a contamination occurs in axenic animals (1.06E+07 to 7.83E+07 EqGen/g).

The dispersal capacity in unoccupied environment theory mentioned above is supported by the fact that using cultures, microscopy and molecular method, the contaminants were detected at very high concentrations after only 4 days. This suggests that when an axenic mouse is contaminated by a microbial agent, the microorganism will colonize the mouse to such an extent that detection is obvious. The limitations for detecting low concentrations of microbes would therefore be problematic for protocols that are shorter than 4 days and not for those that are longer than 4 days.

#### Selecting the Detection Method(s)

Many aspects must be considered in order to choose the most efficient method to detect a contamination. First, according to our results from non-axenic mice and accidental contaminations, the effectiveness of each of the methods used alone was 66%-100% when using culture methods, 80% using microscopy (with a skilled microscopist), and 100% using molecular method. The time required for each technique differed depending on the quantity of axenic samples and animal well-being should be considered. The culture method required up to 7 days of incubation while microscopic results were delivered within 24 h. Molecular method results were available within 2 days. The cost of each method is also an important factor to consider. Microscopy method is considered to be the least expensive method when only a microscope, slides and coverslips are necessary. However, the costs associated with the time required can vary depending on the microscopist’s experience. The culture method is also inexpensive when only considering the price of materials (culture media, tubes), but can be more expensive in circumstances where all the required equipment (incubators and autoclaves, etc.) is not available. The purchase or rental of the necessary equipment can be quite expensive, but in order to ensure profitability, these costs must be factored into the *per diem* price of the mice. The materials required for molecular methods are the most expensive (extraction kit and Mastermix reagents, etc.), not to mention the equipment required to run these analyses, which have major implications for the overall price. Another factor that should be considered when choosing a detection method is the frequency of analysis. The frequency at which quality control is conducted for a certain microorganism should be representative of its likelihood of entering into the cages. The literature suggests that most of the contaminants detected in axenic animal facilities are human skin colonizer and environmental microorganisms (Basic and Bleich, 2019; Lebeuf et al., 2021). According to researchers, the risk of viruses and parasites entering into the cages is very low (Nicklas et al., 2015; Basic and Bleich, 2019), and consequently, they require quality control detection less often than bacteria and environmental microorganisms and *Archaea*, which might also colonize axenic animals (Samuel and Gordon, 2006; Ramezani et al., 2018). Nicklas et al. (2015) have published interesting data about the frequency of analysis in axenic animal facility isolators. They proposed methods and testing frequencies depending on the sample type, which in this case were swabs of the isolator. PCR and culture methods were used to analyze these samples before animals were introduced into the isolator and control testing of the bedding and old food was conducted every 4 weeks. Multiple types of analyses from animal necropsy samples were performed every 3 to 6 months (microscopy, culture, virology, parasitology, and PCR), and culture and PCR of fecal samples were performed every 4 weeks or before and during the transfer of animals. Finally, indicator tests for autoclave sterility were conducted for every batch of water, bedding and food (Nicklas et al., 2015). The use of cage level isolator (Isocage) in our study, which is more at risk of microbial contamination than the traditional, suggests that the frequency of testing applied by Nicklas et al. could be altered, given that each Isocage can harbor unique contaminants that other cages in the same rack might not contain.

When using Isocage or similar models, we recommend : decontaminating the station surface (IBS) with biological controls for every protocol; controlling one cage at each sterilization batch (including the bedding, food, and water) using culture and biological controls; and controlling the interior of the cage every time it is opened using at least two different methods (to screen for as many microorganisms as possible). We suggest the use of a confirmation method to help determine the presence or absence of a microorganisms, as has been advised by other authors (Packey et al., 2013; Nicklas et al., 2015). If it is necessary to open the cage often (e.g., every day during a protocol), we would recommend screening for contaminants at the beginning of the protocol and then again every 4 days (since after 4 days, all the samples will be greatly positive according to most of the tested here). However, because a detection method for fungi, parasites, *Archaea*, and viruses has not yet been developed for Isocage, we cannot provide recommendation for their screening frequency.

## Conclusion

This study has proposed simple ways to detect contaminants in axenic facilities using Isocage type isolators. These approaches were developed through the validation and optimization of three different methods (culture, microscopy, and molecular) as well as by challenging these three methods *in situ* by spiking axenic animals with various microorganisms. The study results suggest that feces can be used exclusively to detect contaminants in axenic animal facility for microbiological quality control purpose with microscopy (wet mounts) and molecular methods (quantitative PCR). For the culture method, feces and surface station (IBS) swabs in Brain Heart Infusion for 7 days (25°C and 37°C in aerobic conditions, and at 30°C in anaerobic conditions) were recommended to detect contaminants. *In situ* results suggests that the culture and molecular methods can detect up to 100% of bacterial contamination events while the microscopy approach generates many erroneous results when not performed by a skilled microscopist. *In situ* results also suggest that when an axenic mouse is contaminated by a microbial agent, the microorganism will colonize a mouse to such an extent that detection is obvious in 4 days, in average. The detection method recommendations when using Isocage type isolator were provided based on : the effectiveness of the tested methods to detect spiked and natural contaminants, the different microbial types screened by each method, the time and cost associated with each method, and the frequency of analyses and finally animal well-being. This report validates simple but complementary tests that can be used by Isocage type isolator users to have an optimal detection of contaminants in axenic animal facilities using. The same procedures could be applied for the development of detection method for fungi, parasites, *Archaea* and viruses, which has not yet been developed for Isocages. The food industry targeting several fungi by routine to ensure costumer health (El Sheikha et al., 2018), their detection methods could be a great starting point to develop quality control procedures in axenic mice facilities.

## Data Availability Statement

The raw data supporting the conclusions of this article will be made available by the authors, without undue reservation.

## Ethics Statement

The animal study was reviewed and approved by Comité de Protection des Animaux de l’Université Laval (CPAUL) and Conseil Canadien de Protection des Animaux (CCPA).

## Author Contributions

ML, NT, CF, ÉP, and CD contributed to conception and design of the study. JR and AM contributed to the conception of the work. ML, CF, and AP contributed to the acquisition of data for the work. ML contributed to the analysis and interpretation of the data for the work and wrote the first draft of the manuscript. All authors revised critically the manuscript for important intellectual content, read the work, and approved the submitted version.

## Conflict of Interest

AM research chair was given in partnership between IRSC and Pfizer Canada and he is consequently not employed by Pfizer Canada. The remaining authors declare that the research was conducted in the absence of any commercial or financial relationships that could be construed as a potential conflict of interest.

## Publisher’s Note

All claims expressed in this article are solely those of the authors and do not necessarily represent those of their affiliated organizations, or those of the publisher, the editors and the reviewers. Any product that may be evaluated in this article, or claim that may be made by its manufacturer, is not guaranteed or endorsed by the publisher.

## References

[B1] Armbruster D. A. Pry T. (2008). Limit of blank, limit of detection and limit of quantitation. *Clin. Biochem. Rev.* 29(Suppl. 1) S49–S52. 18852857 PMC2556583

[B2] Arvidsson C. Hallén A. Bäckhed F. (2012). Generating and analyzing germ-free mice. *Curr. Protoc. Mouse Biol.* 2 307–316. 26069017 10.1002/9780470942390.mo120064

[B3] Atlas R. M. Bartha R. Atlas D. (1998). *Microbial Ecology: Fundamentals and Applications.* San Francisco, CA: Benjamin/Cummings.

[B4] Bach H. J. Tomanova J. Schloter M. Munch J. (2002). Enumeration of total bacteria and bacteria with genes for proteolytic activity in pure cultures and in environmental samples by quantitative PCR mediated amplification. *J. Microbiol. Methods* 49 235–245. 10.1016/S0167-7012(01)00370-0 11869788

[B5] Basic M. Bleich A. (2019). Gnotobiotics: past, present and future. *Lab. Anim.* 53 232–243. 10.1177/0023677219836715 31096878

[B6] Blais Lecours P. Veillette M. Marsolais D. Duchaine C. (2012). Characterization of bioaerosols from dairy barns: reconstructing the puzzle of occupational respiratory diseases by using molecular approaches. *Appl. Environ. Microbiol.* 78 3242–3248. 10.1128/aem.07661-11 22367078 PMC3346481

[B7] Brielmeier M. Mahabir E. Needham J. R. Lengger C. Wilhelm P. Schmidt J. (2006). Microbiological monitoring of laboratory mice and biocontainment in individually ventilated cages: a field study. *Lab. Anim.* 40 247–260. 10.1258/002367706777611497 16803642

[B8] Catanzaro R. Anzalone M. G. Calabrese F. Milazzo M. Capuana M. L. Italia A. (2015). The gut microbiota and its correlations with the central nervous system disorders. *Panminerva Med.* 57 127–143. 25390799

[B9] Clark R. A. (2017). Barrier busting yeast brew trouble in the gut. *Sci. Immunol.* 2:eaan2237. 10.1126/sciimmunol.aan2237 28738019

[B10] El Sheikha A. Levin R. Xu J. (2018). *Molecular Techniques in Food Biology: Safety, Biotechnology, Authenticity & Traceability.* Hoboken, NJ: John Wiley & Sons Ltd.

[B11] El Sheikha A. F. (2019). Molecular detection of mycotoxigenic fungi in foods: the case for using pcr-dgge. *Food Biotechnol.* 33 54–108. 10.1080/08905436.2018.1547644

[B12] Fontaine C. A. Skorupski A. M. Vowles C. J. Anderson N. E. Poe S. A. Eaton K. A. (2015). How free of germs is germ-free? Detection of bacterial contamination in a germ free mouse unit. *Gut Microbes* 6 225–233. 10.1080/19490976.2015.1054596 26018301 PMC4615677

[B13] Furusawa Y. Obata Y. Fukuda S. Endo T. A. Nakato G. Takahashi D. (2013). Commensal microbe-derived butyrate induces the differentiation of colonic regulatory T cells. *Nature* 504 446–450. 10.1038/nature12721 24226770

[B14] Hartmann P. Chu H. K. Duan Y. Schnabl B. (2019). Gut microbiota in liver disease: too much is harmful, nothing at all is not helpful either. *Am. J. Physiol. Gastrointest. Liver Physiol.* 316 G563–G573. 10.1152/ajpgi.00370.2018 30767680 PMC6580239

[B15] Hecht G. Bar-Nathan C. Milite G. Alon I. Moshe Y. Greenfeld L. (2014). A simple cage-autonomous method for the maintenance of the barrier status of germ-free mice during experimentation. *Lab. Anim.* 48 292–297. 10.1177/0023677214544728 25097255

[B16] Itoh K. Mitsuoka T. (1985). Characterization of clostridia isolated from faeces of limited flora mice and their effect on caecal size when associated with germ-free mice. *Lab. Anim.* 19 111–118. 10.1258/002367785780942589 3889493

[B17] Just N. Lecours P. B. Marcoux-Voiselle M. Kirychuk S. Veillette M. Singh B. (2013). Archaeal characterization of bioaerosols from cage-housed and floor-housed poultry operations. *Can. J. Microbiol.* 59 46–50. 10.1139/cjm-2012-0305 23391229

[B18] Karaman M. (2015). Microbiological standardization in small laboratory animals and recommendations for the monitoring. *J. Clin. Anal. Med.* 6 673–677. 10.4328/jcam.2195

[B19] Lahiri S. Kim H. Garcia-Perez I. Reza M. M. Martin K. A. Kundu P. (2019). The gut microbiota influences skeletal muscle mass and function in mice. *Sci. Transl. Med.* 11:15. 10.1126/scitranslmed.aan5662 31341063 PMC7501733

[B20] Lange M. E. Uwiera R. R. E. Inglis G. D. (2019). Housing gnotobiotic mice in conventional animal facilities. *Curr. Protoc. Mouse Biol.* 9:e59. 10.1002/cpmo.59 30645047

[B21] Lebeuf M. Turgeon N. Faubert C. Robillard J. Paradis E. Duchaine C. (2021). Managing the bacterial contamination risk in an axenic mice animal facility. *Can. J. Microbiol.* 10.1139/cjm-2020-0519 33844954

[B22] Marchesi J. R. Sato T. Weightman A. J. Martin T. A. Fry J. C. Hiom S. J. (1998). Design and evaluation of useful bacterium-specific PCR primers that amplify genes coding for bacterial 16S rRNA. *Appl. Environ. Microbiol.* 64 795–799. 10.1128/AEM.64.2.795-799.1998 9464425 PMC106123

[B23] McArthur J. V. (2006). *Microbial Ecology: An Evolutionary Approach.* Amsterdam: Elsevier Science.

[B24] Midtvedt T. Gustafsson B. (2005). Digestion of dead bacteria by germ-free rats. *Curr. Microbiol.* 6 13–15.

[B25] Nicklas W. Keubler L. Bleich A. (2015). Maintaining and monitoring the defined microbiota status of gnotobiotic rodents. *ILAR J.* 56 241–249. 10.1093/ilar/ilv029 26323633

[B26] Niimi K. Hardy P. Bilecki B. Takahashi E. (2019). Rearing and breeding of germ-free mice for over 1 year in a sealed positive pressure cage system. *Jpn. J. Vet. Res.* 67 119–125. 10.14943/jjvr.67.1.119

[B27] Niimi K. Takahashi E. (2019). New system to examine the activity and water and food intake of germ-free mice in a sealed positive-pressure cage. *Heliyon* 5:e02176. 10.1016/j.heliyon.2019.e02176 31463382 PMC6706585

[B28] Packey C. D. Shanahan M. T. Manick S. Bower M. A. Ellermann M. Tonkonogy S. L. (2013). Molecular detection of bacterial contamination in gnotobiotic rodent units. *Gut Microbes* 4 361–370. 10.4161/gmic.25824 23887190 PMC3839980

[B29] Paik J. Pershutkina O. Meeker S. Yi J. J. Dowling S. Hsu C. (2015). Potential for using a hermetically-sealed, positive-pressured isocage system for studies involving germ-free mice outside a flexible-film isolator. *Gut Microbes* 6 255–265. 10.1080/19490976.2015.1064576 26177210 PMC4615381

[B30] Ramezani A. Nolin T. D. Barrows I. R. Serrano M. G. Buck G. A. Regunathan-Shenk R. (2018). Gut colonization with methanogenic archaea lowers plasma trimethylamine n-oxide concentrations in apolipoprotein e-/- mice. *Sci. Rep.* 8:11. 10.1038/s41598-018-33018-5 30283097 PMC6170401

[B31] Ridaura V. K. Faith J. J. Rey F. E. Cheng J. Y. Duncan A. E. Kau A. L. (2013). Gut microbiota from twins discordant for obesity modulate metabolism in mice. *Science* 341 1079–U1049. 10.1126/science.1241214 24009397 PMC3829625

[B32] Robin J. D. Ludlow A. T. Laranger R. Wright W. E. Shay J. W. (2016). Comparison of dna quantification methods for next generation sequencing. *Sci. Rep.* 6:24067. 10.1038/srep24067 27048884 PMC4822169

[B33] Samuel B. S. Gordon J. I. (2006). A humanized gnotobiotic mouse model of host-archaeal-bacterial mutualism. *Proc. Natl. Acad. Sci. U.S.A.* 103 10011–10016. 10.1073/pnas.0602187103 16782812 PMC1479766

[B34] Schoeb T. R. Rahija R. J. Boyd C. Orcutt R. P. Eaton K. A. (2017). “Chapter 2–principles of establishing and operating a gnotobiotic facility,” in *Gnotobiotics*, eds Schoeb T. R. Eaton K. A. (Cambridge, MA: Academic Press), 21–63.

[B35] Taylor D. M. Read L. Neal D. L. (1986). Determining the viability of fecal bacteria present in germ-free mice. *Lab. Anim.* 20 22–26. 10.1258/002367786781062106 3512905

[B36] Tlaskalova-Hogenova H. Stepankova R. Kozakova H. Hudcovic T. Vannucci L. Tuckova L. (2011). The role of gut microbiota (commensal bacteria) and the mucosal barrier in the pathogenesis of inflammatory and autoimmune diseases and cancer: contribution of germ-free and gnotobiotic animal models of human diseases. *Cell. Mol. Immunol.* 8 110–120. 10.1038/cmi.2010.67 21278760 PMC4003137

[B37] White, Bruns T. Lee S. Taylor J. (1990). “Amplification and direct sequencing of fungal ribosomal RNA genes for phylogenetics,” in *PCR Protocols: A Guide to Methods and Applications*, eds Innis M. A. Gelfand D. H. Sninsky J. J. White T. J. (San Diego, CA: Academic Press), 315–322.

